# Unusual pattern of inferior vena cava thrombosis after veno-arterial extracorporeal membrane oxygenation: a report of two cases

**DOI:** 10.3325/cmj.2020.61.556

**Published:** 2020-12

**Authors:** Marin Pavlov, Zdravko Babić, Nikola Bulj, Tomislav Krčmar

**Affiliations:** 1 Department of Cardiology, Sestre Milosrdnice University Hospital Center Zagreb, Zagreb, Croatia; 2 University of Zagreb, School of Medicine, Zagreb, Croatia

## Abstract

We report two cases of inferior vena cava (IVC) thrombosis after the retrieval of veno-arterial extracorporeal membrane oxygenation cannulas. In both patients, the venous cannula tips were placed in the upper half of the right atrium, enabling adequate blood drainage. During support, uneventful periods of IVC collapse were detected. After decannulation, thrombotic formations resembling a mold of the venous cannula were detected in both patients. Whether the IVC collapse caused IVC thrombosis during VA-ECMO support remains to be determined in further trials.

In the recent decade, veno-arterial extracorporeal membrane oxygenation (ECMO) has gained extensive acceptance in critical care. The method is still burdened by a substantial number of complications. Thrombosis of the veins distal to the ECMO cannula implantation site is a well known complication. However, studies suggest that inferior vena cava (IVC) thrombosis in this setting is underestimated (1). We report two cases of an unusual pattern of IVC thrombosis.

## Case 1

**Figure 1:**
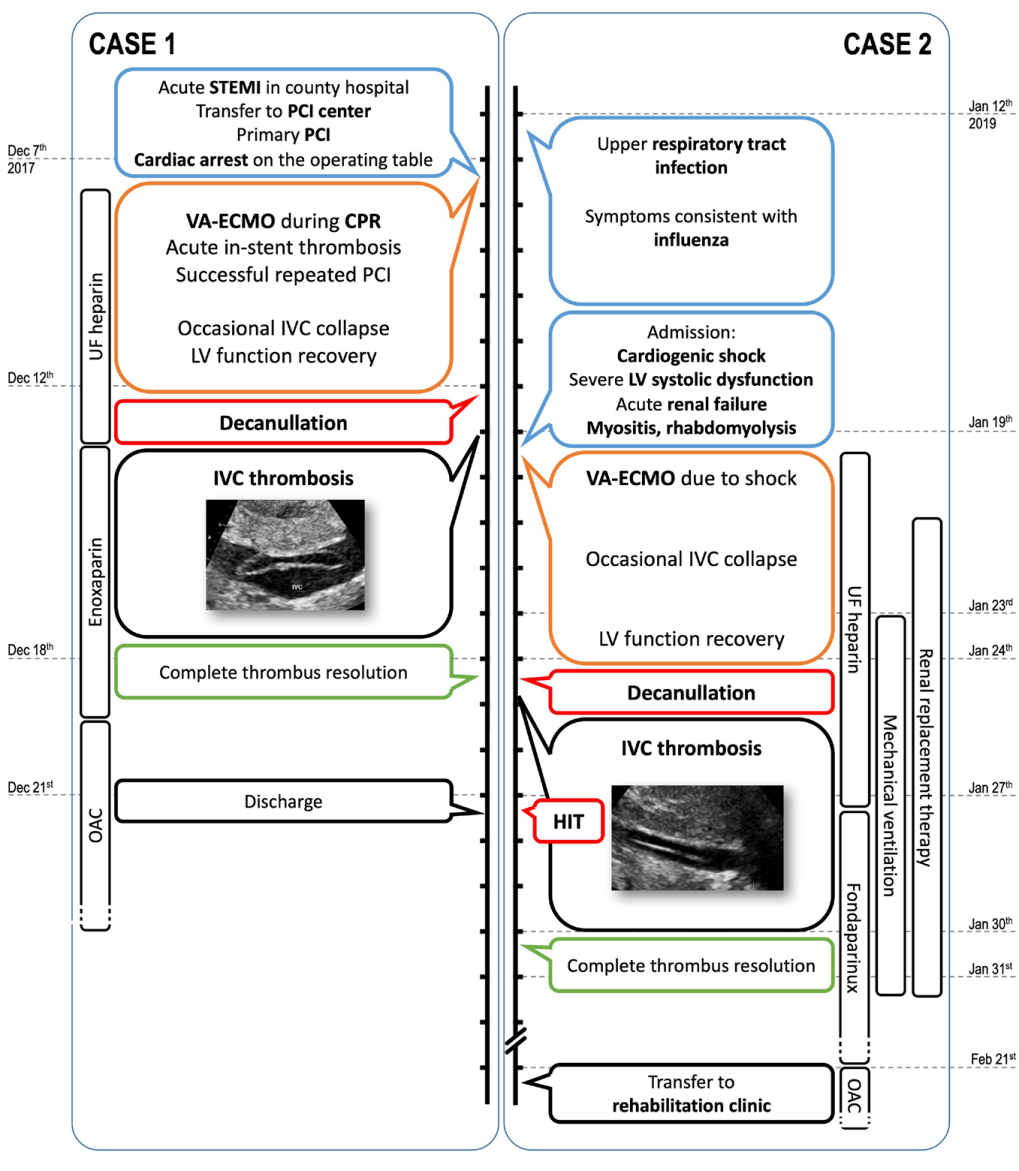
Timeline for both cases. CPR – cardiopulmonary resuscitation, HIT – heparin-induced thrombocytopenia, IVC – inferior vena cava, LV – left ventricle, OAC – oral anticoagulant, PCI – percutaneous coronary intervention, STEMI – ST elevation myocardial infarction, UF – unfractionated, VA-ECMO – veno-arterial extracorporeal membrane oxygenation.

**Figure 2:**
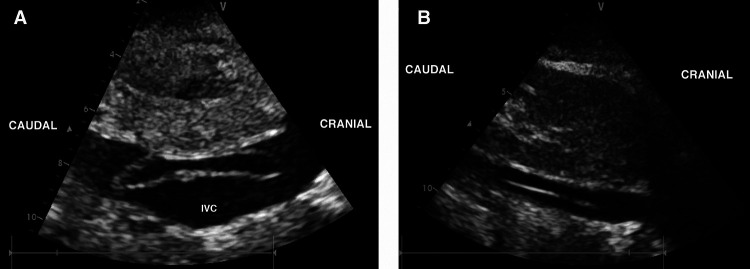
(**A**) Tubular formation with hyperechogenic walls, free floating distal (left side of the figure) and fixed proximal part (right side of the figure), attached to the anterior wall of the inferior vena cava (IVC) (case 1). (**B**) Thin, hyperechogenic filamentous structure attached to the anterior wall of the inferior vena cava (case 2).

A 57-year-old man with a history of smoking, otherwise healthy, was admitted to the emergency department (ED) of a county hospital for chest pain and dyspnea at 9:11 on December 7, 2017 (Figure 1). Rapid workup revealed acute anterior ST elevation myocardial infarction complicated by heart failure. Dual antiplatelet regimen with aspirin and ticagrelor was started. Immediate transfer to our hospital's percutaneous coronary intervention (PCI) center was arranged. The patient arrived at 10:15. An urgent PCI using transfemoral approach revealed occlusion of the proximal left anterior descendent artery as a culprit lesion. Unfractionated heparin iv (100 U/kg) was used. After balloon predilatation, a stent was placed, thus restoring normal coronary blood flow at 10:32. Cardiac arrest ensued promptly, with pulseless electrical activity, refractory to 10-minute resuscitation. Repeated angiogram showed acute stent thrombosis. During resuscitation, percutaneous veno-arterial (VA)-ECMO cannulation in the femoral region was performed (venous cannula 23F/55 cm, tip in the upper half of the right atrium; arterial cannula 17F/15 cm, support initiated at 10:59). A repeated PCI was successful, restoring normal coronary blood flow at 11:18. Continuous iv unfractionated heparin was used to achieve activated partial thromboplastin time (APTT) over 60 seconds (lower than usual due to dual antiplatelet therapy). Support was maintained at 3.0 to 3.5 L/min. Occasional IVC collapse was detected by bedside echocardiography, however, no venous drainage issues were encountered. Left ventriclular (LV) function gradually improved. On December 12, successful decannulation was performed. After decannulation, a long tubular, dual-lining, floating formation, attached to IVC wall 10 mm distal to the IVC-right atrium junction, was observed by echocardiography (Figure 2A, Video 1(supplementary video)). Enoxaparine 80 mg twice a day was initiated. The formation dissolved gradually, with complete resolution on December 18. The patient was discharged on December 21 to cardiac rehabilitation in good neurologic (cerebral performance category I) and physical condition, and has been free from thrombotic and thromboembolic incidents since.

## Case 2

A 39-year-old woman with no chronic medical condition acquired a febrile upper respiratory tract infection consistent with influenza on January 12 (Figure 1). The patient's mother (living in the same household) was home treated for influenza since January 6, 2019. On January 19 at 9:00, the patient presented to our hospital's ED with light-headedness, dyspnea, and tachypnea. On examination, tachycardia (122/min), hypotension (82/50 mm Hg), tachypnea (27/min), and signs of hypoperfusion (cold extremities, mottled, clammy skin) were present. The initial laboratory findings revealed positive markers of cardiac myonecrosis (high-sensitivity troponin I 3370 ng/L). Urgent echocardiography showed a normal-sized LV with symmetric wall hypertrophy (septum 21 mm) and ejection fraction of 20%. The diagnosis of cardiogenic shock due to influenza myocarditis was established. The initial approach with volume, dobutamine, and norepinephrine proved ineffective. Due to persistent hypotension and hypoperfusion (oliguria, altered mental status, rise in arterial lactate to 12.0 mmol/L), VA-ECMO support was initiated at 12:35 on the same day (venous cannula 23F/55 cm in the right femoral region, tip in the upper half of the right atrium; arterial cannula 15F/15 cm in the left femoral region). The initial support of 4.0 L/min was reduced to 3.0 L/min on January 20. The disease course was complicated by severe viral myositis, rhabdomyolysis (peak creatine kinase 93.727 U/L), and acute renal failure requiring continuous renal replacement therapy until January 31. Initial heparin (APTT targeted at 70 to 80 seconds) was substituted by fondaparinux (5.0 mg iv once a day; subcutaneously after withdrawal of renal replacement therapy) on January 27 due to heparin-induced thrombocytopenia (HIT). No pulmonary congestion occurred, however, mechanical ventilation was required due to respiratory muscle failure from January 23 until January 31. LV function completely recovered on January 24. Same-day decannulation ensued. Following decannulation, a long dual-lining formation attached to the IVC wall was detected, resembling a cannula mold (Figure 2B, Fondaparinux treatment led to gradual disolution of the formation, with a complete resolution on January 30. Acquired and hereditary thrombophilias other than HIT were ruled out. On February 21, the patient was transferred to a rehabilitation facility with moderate left-sided peroneal paresis, otherwise completely recovered.

## Discussion

VA-ECMO provides lifesaving mechanical cardiac support during acute devastating cardiac conditions, with a potential to serve as a bridge to recovery of LV function and decannulation within days. In the first days of VA-ECMO support, it is of utmost importance to accomplish adequate perfusion while maintaining optimal LV unloading conditions and antegrade aortic systolic flow. Placing the venous cannula tip in the upper part of right atrium or superior vena cava, as suggested by Ruggeri et al (2), allows optimal venous blood drainage. However, there can occur an occasional or persistent IVC collapse, a benign phenomenon outside ECMO support. If not systematically assessed by echocardiography or if not of sufficient magnitude to cause cavitation and hemolysis, the collapse can remain undetected. We believe such IVC collapses were responsible for thrombus formation on the outer venous cannula surface, which persisted as a cannula sleeve thrombus (predominantly fibrinous according to echocardiography) after cannula withdrawal. Since the thrombus was attached to the IVC wall substantially distal to the cannula tip, other explanations for the thrombus formation are highly unlikely. Notably, both patients experienced additional thrombotic events (hyperacute in-stent thrombosis; heparin-induced thrombocytopenia). The reported incidence of venous thrombosis in VA-ECMO cohort is 10% (3). While cases of molding cannula thrombus (4) or of thrombi originating in the cardiac chambers (5) have been described, to our knowledge, a persisting thrombus build-up on the outer cannula wall has not been reported.

IVC collapse during VA-ECMO is a phenomenon of unknown significance regarding issues other than venous drainage. Related complication rates and outcomes have not been systematically investigated. Perforations on the tip of the multi-stage venous cannula drain the majority of the venous return, leaving the caudal part of IVC free of blood flow, and thereby heparin-depleted. Mid-cannula perforations apply negative pressure directly to the venous endothelium, possibly causing trauma and at the same time preventing the IVC from expanding. In this scenario, all three components of the Virchow’s triad are uniquely present in the collapsed part of the IVC. The prerequisites for thrombus build-up at the IVC wall are established, as happened in the presented cases.

In both cases, the thrombus resolution was complete. Given the size of the detected thrombi, complete embolization could have resulted in a major hemodynamic deterioration.

Preventive measures, and regular and systematic assessments of thrombotic complications should be applied in all ECMO patients. Whether a more active approach to avoid IVC collapse is to be introduced remains to be assessed by further clinical investigations. Applying additional iv fluids or blood products if indicated, temporarily lowering pump flow rate, establishing short-term Trendelenburg position, or excluding flow obstruction within the system, is usually suggested to prevent cannula “chattering”. These measures will also promote IVC expansion. Whether this will decrease the occurrence of the described thrombotic events remains a matter of speculation.
